# Women's experience of unexpected caesarean section birth in Kitui County, Kenya

**DOI:** 10.4314/ahs.v23i2.75

**Published:** 2023-06

**Authors:** Zipporah K Kimanthi, Lister N Onsongo

**Affiliations:** Department of Community & Reproductive Health Nursing, School of Nursing Sciences, Kenyatta University

**Keywords:** Birth experiences, midwifery, unexpected CS

## Abstract

**Background:**

In sub-Saharan Africa, 72% of all Caesarean section (CS) births are unplanned compared to 27% of unplanned CS births done in developed countries. Various researches have been conducted on lived experiences following unexpected CS birth but none in Kitui County, Kenya.

**Objective:**

This study described the lived experience of undergoing an unexpected CS and the role of cultural beliefs on childbirth among Kitui/Kamba women in Kenya.

**Methods:**

A descriptive phenomenology design was used in this study. In-depth interviews of women who experienced unplanned Caesarean birth in Kitui County, Kenya, were conducted. Colaizzi's method guided the analysis. Interviews were approximately 30 minutes long and audiotaped.

**Results:**

A total of 12 mothers participated in the study. Fives themes and 11 sub-themes emerged from this study: fear (fear of disability and surgical complications), pain (physical and psychological pain), less of a woman (lowered self-esteem, powerless and worry), sullied (dispirited and will loss), and fallacy (misconception and effects of fallacy).

**Conclusion:**

Disruption, dissatisfaction with the birth process, and unmet expectations were negative experiences. Healthcare workers should be sensitive when informing mothers of unplanned CS. More research to look for coping strategies to reduce negative birthing experiences.

## Introduction

Caesarean section (CS) accounts for 18.5 million births globally 1. The Maternal Mortality Rate due to CS is 100 times higher in developing countries 2. Annually, unexpected CS account for more than 80% of all the CS done globally. Most (85%) of CS births in the United States are unplanned 3. Caesarean section rates have risen in all regions since 1990 and continue to increase globally, averaging from 5% in sub-Saharan Africa to 43% in Latin America 3.

Without effective global interventions to revert the trend, Southern Asia and sub-Saharan Africa will face a complex scenario due to the overuse of resources for surgical procedures. This trend will lead to increased morbidity and mortality associated with unmet needs and unsafe provision for CS 4. In Sub-Saharan Africa, CS births in private hospitals account for 72% of all births 5. A study done in Nigeria by 6 revealed that 90% of CS births were unexpected. Mothers who undergo CS birth while visualizing what is going on have a better birthing experience^7^. Childbirth and passage to motherhood through a Normal Vaginal Birth (NVB) make a woman feel unique and have a feeling of being a total woman 8. Women following CS experience incisional pain, reduced mobility, reduced self-care, bonding deficit, prolonged hospital stay, anxiety, post-traumatic stress, and depression 9.

Many women resist unexpected CS for fear of failing in their culturally assigned gender roles which could endanger their marriage. These fears lead to delayed CS birthing consent^10^. Culturally, in Greek, it is believed that idle mothers during pregnancy end up undergoing a CS birth^11^, ^12^. Cases of CS in Kenya have risen from 26% to 44% in the last four years 5 despite the WHO standard of 10-15% 3.

In Kitui County, CS has a rising trend; 11% to 18% from 2017 to 2020 13, 14. The causes of CS in Kenya include fetal and maternal distress, prolonged labour, placenta abruption, and cord prolapse, among others 14. Despite the upsurge of CS cases, the mother's experience following the unexpected Caesarean birth remains a lived reality of feelings, perceptions, and attitudes 1 Various studies on unexpected birthing experiences have been reported globally 15, 4 2 8. However, no such study in Kitui County, Kenya. Therefore, this study aimed to describe the lived experience following unexpected CS birth and the role of cultural beliefs associated with CS among Kamba/Kitui women in Kenya.

## Methods

### Participants and study site

Kitui County was the study site. It has one County referral hospital (KCRH), 14 sub-county hospitals, 56 health centres and 383 dispensaries 15. Mothers who had undergone unexpected CS, with no previous CS, aged 15-49 years and had an outcome of a healthy live baby participated in this study.

### Design

A descriptive phenomenology design was employed in this study. This design strives to reveal and understand the intrinsic nature of a phenomenon 16. Phenomenological knowledge is grounded in the belief that the lived experience is a reality. Through reflection and understanding of the lived experience, knowledge is generated by making sense of the lived experience 17. Phenomenology was used in this study to reveal lived experiences following unexpected CS birth.

### Recruitment and Sampling Techniques

The researcher used a purposive criterion sampling method to recruit 12 participants from the Maternity ward in Kitui County Referral Hospital. Purposive criterion sampling enables the researcher to choose those participants with specific characteristics 18. Thus researcher used this method to select mothers who fit the inclusion criteria. Following ethical approval, a midwife at the KCRH maternity unit recruited participants within three days post-CS by scrutinizing the maternity register, and the mother's admission files/partograph, showing that the labour process was halted for CS was inevitable. The midwife identified, approached and sought permission from a potential participant who met the study inclusion criteria before the researcher entered the birthing suite to meet the participants. After identifying the mothers, the researcher explained the study and the purpose of recruiting them, agreed on when and where to meet for the interviews and exchanged contacts. The investigator contacted the mother a few days before the agreed interview date to confirm any changes, which also served as a reminder. The interview occurred between the sixth week and sixth-month post-CS birth, between August 2020 to February 2021, at MCH of KCRH or the mothers' home. The researcher pardoned her previous knowledge of CS experience and trusted the information the participants gave was accurate, which is evidence of what exists, thus avoiding recall bias. During recruitment and data collection, the researcher was not providing care in these units; hence no conflict of interest.

### Data collection methods

The researcher used a semi-structured interview guide to collect data. Following the participant's consent, the interviews were audiotaped and took an average of 30 minutes. Data collection stopped after reaching saturation. All the 12 women approached by the researcher consented to the study, recruited, and participated. The researcher allowed each participant much freedom to describe their emotions and feelings about the unexpected CS birth.

To ensure the validity of the results, the researcher provided trustworthiness using 19. This model identifies five applied aspects: authenticity, credibility, dependability, conformability & transferability. The first author, a skilled birth attendant, collected and transcribed the data. The second author guided the coding and analysis of the data. Questions were open-ended and elicited experiences related to undergoing an unexpected CS and the role of culture in birth. The questions kept on being modified as per the participant's perception. For example, the researcher asked questions such as;

Tell me about the pregnancy and childbirth experiences; what was that experience like for you? Please include what was meaningful and unhelpful to you during your Caesarean section experience. Help me understand how you received the news of the Caesarean section and what your experiences were?

### Data analysis

The researchers used Colaizzi's method 20 to guide the analysis. The analysis included reading the transcripts multiple times to gain insight into the data's meanings. Significant statements and phrases were classified and restated in broad terms, formulating definitions and validating intentions by the two authors through discussions to agree on themes and sub-themes. They maintained an audit trail to ensure they could trace all steps taken in the analysis to the interviews. They used participants' quotes to establish descriptive validity.

### Ethical approvals

Ethical approvals were obtained from Kenyatta University's Ethics Review Committee, the National Commission for Science, Technology, and Innovation, the Hospital Administrator in charge of MCH, and the maternity unit. Participants gave informed consent before the interview session. The researcher took the participant through the consent procedures to be followed and signed the participant's form of consent that she understood. During the interview process, any complaint from the participants was referred accordingly.

## Findings

### Participants Characteristics

The average age of women was 28 (±7) years. Most (67%) participants were married and had attained secondary and tertiary education (84%). Most (73%) of women were employed, had one previous vaginal birth (58%), and the majority (92%) were Christians (Table 4.1).

**Table 4.1:** Participants' characteristics

Variable	Category	Frequency (N=12)	Percentage
Age	Participants' 15-49 years	12	100%
Marital status	Married	8	67%
	Single	3	25%
	Divorced	1	8%
Education level	Primary	2	16%
	Secondary	5	42%
	Tertiary	5	42%
Employed	Yes	9	75%
	No	3	25%
Number of previous births	NVB	< 2 Births 7	58%
		> 2 births 5	42%
Religion	Christianity	11	92%
	Islamic	1	8%

### Themes

The researchers grouped the findings into five themes and 11 sub-themes from a total of 12 participants: Theme 1) Fear (disability and surgical complications), 2) Pain (physical and psychological), 3) Less of a woman (lowered self-esteem, powerless and worry), 4) Sullied expectations (dispirited and will loss), 5) Fallacy (misconception and effects of fallacy) (Table 4.2).

**Table 4.2:** Emergence of Themes

Cluster of themes	Sub Themes	Examples
Fear	Fear of Disability	*“…I feared the numbness of the legs caused by the theatre drugs will make me unable to turn on bed, walk, bath, feed baby” (Participant 6)*
	Fear of surgical complications	*“… I feared instruments may be left in my stomach, and I may never conceive again” (participant 3)*
Pain	Physical pain	*“On my 6th week, the pain continues up to now, I still feel pain, I can't even fulfill my conjugal rights (Participant 2)*

	Psychological pain	*“I feared the pain I will experience on the cut wound following the CS will be too much.” (Participant 12)*
Less of a woman	Lowered self esteem	*“… I questioned my abilities for failing to push, I fe like a lesser woman” (Participant 1)*

	Powerless	*“I felt have failed like a woman, not undergoing labor successfully stressed me and made feel that I had no power of a real woman” (Participant 12)*
	Worry	*“… now being told I will be done CS I got worried, and wondered what could have happened and the previous birth was normal” (Participant 7)*
Sullied expectations	Dispirited	*“my expectations were tarnished by the CS news” (Participant 2)*
	Will loss	*“I wanted to have a normal birth, of short duration …as a result, I did not expect to be taken for the CS.” (Participant 3)*
Fallacy	Misconception	*“…my community associates CS with evil deeds, for the mother, baby or both may die during the operation…” (Participant 11)*
	Effects of Fallacy	*“some community people belief CS is a procedure for the modern generation to preserve their birth canal for future sexual pleasure” (Participant 7)*

### Theme 1: Fear

Most mothers indicated that they feared a CS birth because of their unpreparedness and the unexpected outcome of the procedure based on the narratives they had heard in their social circles.

### Fear of disability

Almost all the mothers in the study consistently reported not being able to undertake their activities of daily living following CS. Their fear was due to a lack of prior preparations for the anticipated experience as they narrated:

“*I had some difficulties in attending to my normal duties, like walking to the toilet, holding the baby to breastfeed, changing position in bed, bathing, for real, I was unable to support myself physically*” (Participant 7).

### Fear of surgical complications

Eleven mothers feared CS birth due to possible surgical complications. Most of them said they experienced intense fear after being told they would undergo CS birth. Women attributed the fear of surgery to discussions they had had with their families or peers as they narrated:

“I feared that I may fail to wake up from the operation table, my baby can be exchanged while am under the sleeping drug in theatre, instruments may be left in the stomach, I may be transfused, and the blood may be infected with HIV” (Participant 7).

“*I feared that I may fail to wake up from the operation bed and may bleedto death*” (Participant 9).

### Theme 2: Pain

Ten women reported CS as a painful experience; physical and psychological pain experienced after surgery and discharge from the hospital can be debilitating and disabling, based on their narratives:

“… *after the numbness was over, the pain on the surgical site was too much more than I anticipated, I was unable to hold the baby and breastfeed, walk to the toilet, pass stool so I ate sparingly, because of the pain*” (Participant 6).

### Theme 3: Less of a woman

Participants talked about social and cultural perceptions' influence on women who undergo a CS birth. Social and cultural expectations such as a woman's role in the household were impossible; they felt they had failed in their gender roles, as reported:

“*CS has robbed me the feeling of a real woman, I feel embarrassed and disappointed for not representing women's power and ability in childbirth*” (Participant 6).

### Theme 4: Sullied expectations

The results show that almost half of the mothers did not expect to undergo CS, they thought their normal pregnancy period was a high way to Normal Vaginal Birth, but the reverse happened, based on their narratives:

“*I thought I will undergo normal birth, be fine, get a healthy baby and most probably go back to my normal chores immediately, but the same never happened*” (Participant 4).

### Theme 5: Fallacy

Ten mothers attributed CS to mistaken beliefs of evils deeds, idleness, grudges or prestigious birth mode as they quoted:

#### Sub Theme: Misconception

“*… Someone has been bewitched, so you need traditional cleansing to overcome the evil intentions on you for labor process to be normal*” (Participant 11).

“… *when you do not work during the period of pregnancy, you will have no efforts to push the baby out, and the baby has assumed your idleness thus fails to come out for it feels it's in its best place*” (Participant 5).

#### Sub Theme: Effects of fallacy

Eleven mothers expressed embarrassment and disappointment after mentioning CS and articulating the beliefs and misconceptions surrounding CS.

“*Those who undergo CS are believed to be failures and cowards, unable to face the reality of a real woman*” (Participant 12).

## Discussion

To our knowledge, this is the first study that has explored experiences of undergoing unexpected CS by mothers in Kitui County. At the core of the experience of mothers following unexpected CS, negative effects were identified among these mothers. The mothers reported they could not breastfeed their newborn or perform activities of daily living independently. This finding is consistent with other studies 21, 9, whereby women expressed compromised independence following CS birth. Our analysis also revealed that mothers experienced fear of surgical complications. Similar to 22 that demonstrated, mothers feared complications and death following an operation from CS birth. This information disagrees with a study done among Swedish women, where 37% reported that CS was the best mode of birth, and the consecutive births, mothers preferred CS mode 12. Another study done in the United States by ^23^ disagrees with the results of this study; it showed that mothers preferred the interventions of unexpected CS to assisted vaginal birth because of lousy birth outcomes.

Less of a woman following CS birth was another experience identified in this research. Mothers experienced lowered self-esteem and incompetence in childbirth. This finding is similar to 6, 11, which showed that CS puts the mothers to lowered self-esteem, feeling like a lesser women and unable to face the reality of a real woman.

The mothers reported that their normal birth process expectations were reciprocated with CS, thus ending with sullied expectations. This study finding agrees with 16, which states that CS makes the mothers not experience what they expected for the birth process.

The mothers mistakenly believed that; the mothers who undergo unexpected CS birth were bewitched or had grudges with someone during their pregnancy and needed cleansing or reconciliation respectively before labour incepts. These findings agree with 10 that CS birth is interpreted as a curse or enemy attack and can be resolved through faith and prayer. Others attributed CS birth to a lack of active lifestyle during pregnancy. These results are similar to 11, 12 which showed that most idle mothers during pregnancy ended up with a CS birth. The unique thing in this study: doctors and midwives have learnt that unexpected CS news has significant negative psychological effects; thus, they have realized a need to curb those effects.

## Conclusion

This study concludes that women from Kitui County experience negative perceptions following unexpected CS birth. These perceptions include; disruption of birth plans, dissatisfaction with the birth process and unmet birth expectations. The researchers recommend; that healthcare workers, including doctors and midwives, should be more sensitive when informing mothers of unexpected CS and identify methods and means of alleviating fear and worry. Mothers' support groups should be developed. Knowledge before CS is envisaged to clear the mothers' doubts and demystify misconceptions and beliefs associated with CS birth. Further research should be done to find coping strategies to reduce negative birthing experiences.
